# Efficacy of topical erythropoietin gel and mouthwash for recurrent aphthous stomatitis in a randomized clinical trial

**DOI:** 10.1038/s41598-026-40440-7

**Published:** 2026-03-18

**Authors:** Mai Talaat Elgendi, Radwa R. Hussein, Nivine Ragy, Nada M. El Hoffy, Nevine H. Kheir El Din

**Affiliations:** 1 Department of Oral Medicine and Periodontology, Faculty of Oral and Dental Medicine, Future University, Cairo, Egypt; 2 Department of Oral Medicine and Periodontology, Faculty of Dentistry, Ain Shams University, Cairo, Egypt; 3 Department of Pharmaceutics and Pharmaceutical Technology, Faculty of Pharmacy, Future University in Egypt, Cairo, 11835 Egypt

**Keywords:** Erythropoietin, IL-2, Recurrent aphthous ulcer, Saliva, Diseases, Drug discovery, Health care, Medical research

## Abstract

Erythropoietin (EPO) has anti-inflammatory, antioxidant, and wound-healing properties that could be valuable in managing Recurrent Aphthous Stomatitis (RAS). This study aimed to evaluate its clinical effectiveness in either gel in orabase or mouthwash, on pain reduction, ulcer healing, recurrence rates, and salivary IL-2 level in patients with RAS. In this randomized clinical trial, 60 patients with RAS were equally divided into 3 groups: EPO oral gel, EPO mouthwash, or a placebo. Ulcer size, the Pain Visual Analog Scale (P-VAS), and salivary IL-2 levels were evaluated at baseline, day 3, and day 7. When compared to a placebo, both EPO formulations significantly decreased pain, ulcer size, and salivary IL-2 levels (*p* < 0.001). With nearly total pain relief, significant ulcer healing, and the lowest post-treatment IL-2 concentration, the EPO gel group showed the greatest improvement. In comparison to mouthwash and a placebo, patients treated with EPO gel also had noticeably longer recurrence-free intervals (median 5 months). No negative effects noted. These findings suggest that EPO gel is a therapeutic option for RAS that is both safe and effective, with the potential to modify the disease in addition to relieving symptoms.

Trial registration: The study was registered at Clinical Trials.gov (NCT06923605) on 11/4/2025.

## Introduction

Recurrent aphthous stomatitis (RAS) is a common disorder marked by ulceration of the oral mucosa, with an incidence rate ranging from 5 to 25%^1^. Based on severity, frequency, and number of episodes, RAS is divided into three different categories: Herpetiform, major, and minor types^2^. Among these classifications, minor aphthous ulcers are the most prevalent, with a prevalence rate of 70–85%^3^.

The exact cause of RAS remains unclear, while numerous factors have been proposed as potential contributors to its etiology, including local conditions, genetic predispositions, deficiencies in nutrition and hematinic substances, cessation of smoking, endocrine and immune system dysfunctions, allergic reactions to food and medications, microbial influences, and psychosomatic disorders such as anxiety, stress, and depression^4^.

Given its crucial role in the Th1-mediated immune response in RAS, interleukin-2 (IL-2) was selected as the biochemical marker in this investigation. The pathophysiology of ulcers is aided by IL-2, which stimulates T lymphocyte activation and proliferation and increases the release of other pro-inflammatory cytokines like IL-1, TNF-α, and IFN-γ^5^. Additionally, previous investigations have shown that the saliva of RAS patients has noticeably higher levels of IL-2 than that of healthy controls, confirming the validity of this non-invasive biomarker for disease activity^5,6^. IL-2 was therefore regarded as a useful and clinically significant endpoint for assessing erythropoietin’s anti-inflammatory action.

Corticosteroids are considered the main line of treatment for oral RAS, but they also lead to severe unwanted side effects and a significant risk of infections^7^. Thus, exploring alternative natural treatment options without or with minimal side effects for RAU is required, as those patients use them for long periods of their lives.

Additionally, several anti-inflammatory and regenerative strategies have been investigated; however, the majority focus more on temporary pain relief and epithelialization than on altering the local immuno-inflammatory environment or prolonging remission. The evidence base is still inconsistent, and barrier and biopolymer therapies, like hyaluronic acid (HA) gels and rinses, improve pain and healing time but have little to no effect on biomarker modulation and recurrence^8,9^.

Although platelet concentrates (PRF/PRP) can help with mucosal repair and provide autologous growth factors, they usually involve handling blood or administering injections, and the evidence supporting them is still in the early stages of development and is of mixed quality for RAS in particular^10^.

Erythropoietin (EPO), on the other hand, is a special pleiotropic protein that simultaneously targets the inflammatory and regenerative components of RAS pathogenesis. In contrast to traditional therapies, EPO increases angiogenesis, decreases apoptosis, and speeds up mucosal repair in addition to suppressing important pro-inflammatory pathways like NF-κB-mediated cytokine release^11–15^. For a chronic condition like RAS, where both symptom control and long-term remission are desired, this dual mechanism is especially beneficial. EPO is a good option for long-term use because topical application prevents systemic exposure and the risks that come with it.

Saliva serves as a significant medium for the diagnosis and monitoring of RAS. Its non-invasive nature has garnered considerable attention in the diagnosis of various conditions over the past decade^16–18^. Consequently, saliva holds greater potential diagnostic value, which is widely acknowledged, and it is poised to assist in the diagnosis of both systemic diseases and oral disorders^19–21^.

Owing to its pivotal role in the local inflammatory response implicated in RAS pathogenesis, IL-2 was chosen as a biomarker. A previous study indicates that local application of erythropoietin can still have immunomodulatory effects at the mucosal surface, even though it was applied topically rather than systemically^22^. Finding trustworthy diagnostic and prognostic markers is crucial for oral health research, according to a recent study^23^. Accordingly, to objectively evaluate the therapeutic impact of erythropoietin in RAS, the current trial investigates interleukin-2 as a non-invasive salivary biomarker.

Therefore, this randomized controlled clinical trial aimed to compare the clinical effectiveness of EPO administered in two different application forms, either gel in orabase or mouthwash, in the management of RAS. The primary outcomes were pain intensity and ulcer size, while secondary outcomes included the recurrence rates and salivary IL-2 level in patients with RAS.

We hypothesized that topical erythropoietin would reduce the size of the ulcer, number of recurrences, pain intensity, number and size of the ulcer, and IL-2 levels in the saliva of patients with RAS compared to the mouthwash and placebo.

## Subjects and methods

### Sample size

The sample size was determined based on the primary outcome of pain reduction measured on a 100-mm Visual Analog Scale (VAS) at Day 7. Estimates were derived from a recent randomized controlled trial evaluating thyme honey for minor aphthous ulcers, which reported a mean pain reduction difference of 0.73 units with a pooled standard deviation of 0.70, corresponding to a standardized effect size (Cohen’s *d*) of approximately 1.04^1^. Assuming a two-sided significance level (α) of 0.05 and a statistical power of 80%, the minimum required sample size was calculated to be 16 participants per group. To account for an anticipated dropout rate of 20%, the sample size was increased to 20 participants per group. Given the three-arm design of the present trial (EPO gel, EPO mouthwash, and placebo), a total of 60 participants (20 per arm) was considered sufficient to achieve the desired statistical power.

### Study design and setting

This is a double-blind, placebo-controlled, 3-parallel-arm, and randomized clinical trial. All subjects were recruited from the outpatient clinics at Ain Shams & Future Universities in Egypt.

### Ethical approval, trial registration, and consent

The ethical committee of the Faculty of Dentistry at Ain Shams University approved the study (FDASU-Rec ID032209). The study was performed per the principles of the modified Helsinki’s code for human clinical studies (Association, 2013), and CONSORT 2010 guidelines for reporting randomized clinical trials. The study was registered prospectively at Clinical Trials.gov (NCT06923605) on 11/4/2025.

All participants received comprehensive verbal and written information regarding the study protocol. Their understanding and consent to participate in the study were verified, and each participant provided written informed consent.

### Randomization and allocation concealment

An independent statistician who was not involved in the trial created a computer-generated block randomization sequence using www.randomizer.org. Group I (EPO gel), Group II (EPO mouthwash), or Group III (Placebo) were the three groups to which participants were assigned in a 1:1:1 ratio. Sequentially numbered, opaque, sealed envelopes (SNOSE) were used to hide the allocation sequence. The central pharmacy prepared and stored these envelopes safely. The next sequentially numbered envelope would be opened by the treating clinician to reveal the group assignment once a participant had been judged eligible and given informed consent.

**Blinding**: The trial was double-blind and placebo-controlled. Complete participant blinding was not possible due to the physical differences between a gel and a rinse. Although they were blinded to whether their formulation contained the active drug (EPO) or was a placebo, participants knew if they were using a mouthwash or a gel. To preserve this blinding, all formulations (active and placebo) had the same look, color, texture, viscosity, and taste.

### Blinded personnel

- **Outcome Assessors**: All clinical measurements, including ulcer size and P-VAS collection, were conducted by clinicians who were completely blinded.
- **Data Analysts**: A fully blinded statistician performed the data analysis.

### Unblinded personnel

- **Dispensing Pharmacist**: An unblinded pharmacist created the formulations in accordance with the randomization schedule.
- **Treating Clinicians**: As they handled the allocation envelopes, the clinicians who gave the participants their interventions were not blinded.

All groups were denoted by coded labels (A, B, and C) for assessors and analysts to preserve blinding. The unblinded pharmacist retained the allocation key, which was not made public until the completion of the final statistical analysis.

### Patient selection and eligibility criteria

#### Inclusion criteria

Participants of both genders, aged between 20 and 40 years. They were classified as systemically healthy patients according to the American Society of Anesthesiologists (ASA I).

Participants aged 20–40 years were enrolled to minimize age-related variations in immunologic function and inflammatory salivary biomarkers, and to reduce the potential confounding influence of systemic comorbidities and pharmacologic therapies on the study outcomes^24–26^.

The ulcer must have developed within the past 24 h in all patients, and its size must be ≥ 4 mm and < 10 mm^8,27^.

#### Exclusion criteria

Smokers, as well as pregnant or breastfeeding women, and those with intellectual disabilities, were not included in this study. Additionally, patients who had a history of using any topical or systemic medications or undergoing steroid therapy within one month before the investigation were also excluded^28^.

#### Patients’ recruitment

Patients were gathered from Future Universities’ and Ain Shams’ outpatient clinics.

Minor RAS was clinically identified as multiple, recurrent, tiny, shallow, irregular ulcers with distinct boundaries and erythematous halos on non-keratinized mucosa and a history of recurrence. The diagnosis of recurrent RAS was supported by the patient’s documented history of at least three episodes of comparable aphthous ulcers during the previous 12 months. Two different skilled oral medicine specialists clinically diagnosed RAS (blinded to allocation).

**Fig. 1:**
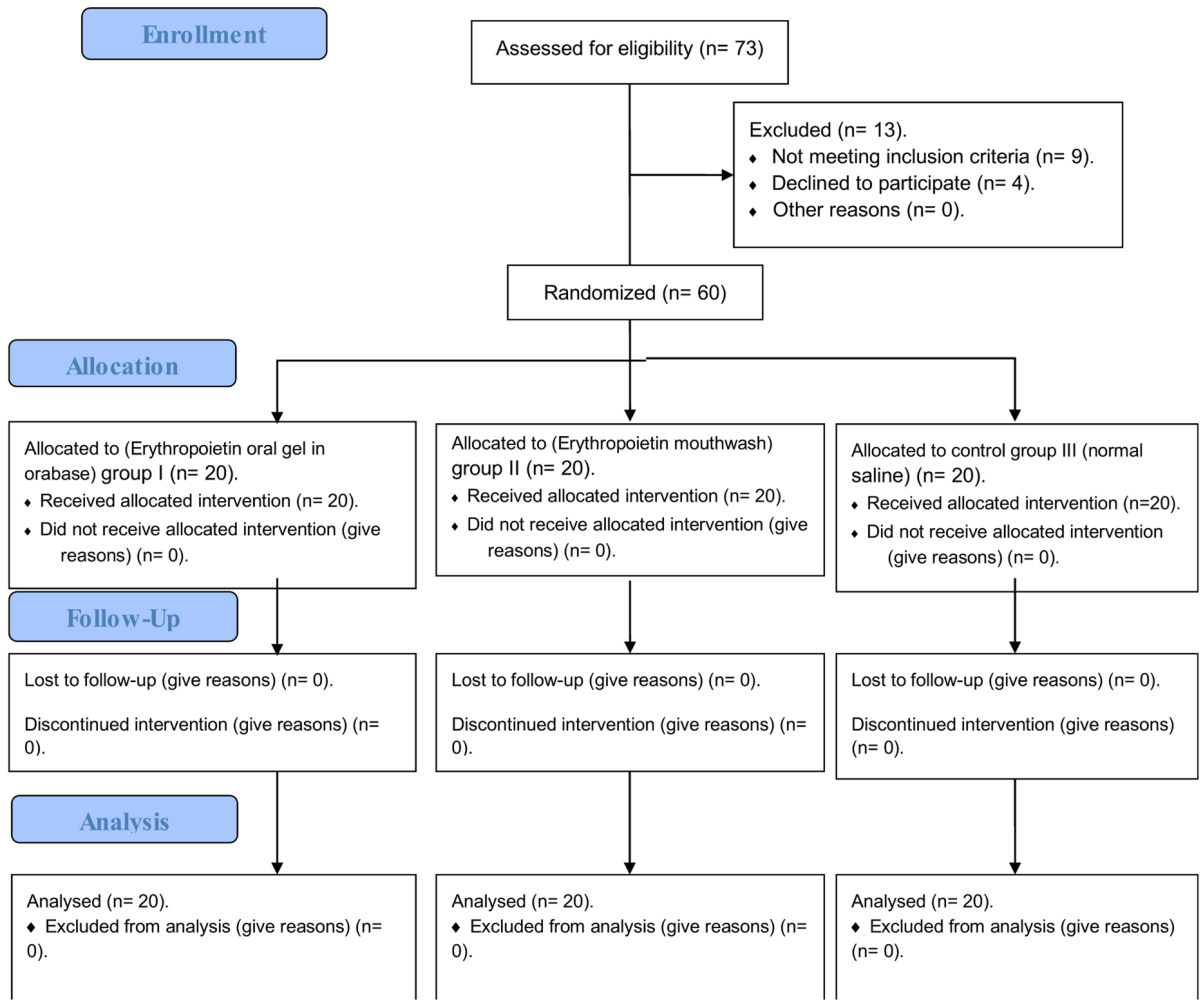
The flow diagram for patients’ recruitment.

Patients who met the eligibility criteria and agreed to participate in the current study were randomly allocated to 3 equal groups with 20 patients in each group. The CONSORT flowchart for patient recruitment and progression is illustrated in Fig. 1.

#### Treatment protocol and patient grouping

**Group I** included 20 patients who received Erythropoietin oral gel in orabase 4 times daily (after meals and at bedtime).

**Group II** included 20 patients who received Erythropoietin mouthwash 4 times daily (after meals and at bedtime).

**Group III** included 20 patients who received the placebo isotonic (normal saline) with a concentration of 0.90% of sodium chloride (NaCl).

### Evaluation of outcomes for all groups

#### Clinical assessment

All clinical assessments, including measurements of ulcer size, were done by a single, highly experienced oral medicine specialist with more than 10 years of experience in clinical work (**M.T).** To ensure that measurements were accurate, a calibration procedure was done before starting the study among volunteer patients using a standardized protocol with a calibrated periodontal probe. The reason for this was to ensure that a high level of measurement reliability was maintained.

**(1) Pain Visual Analog Scale (P-VAS)**^1^

Pain intensity was measured on a Visual Analog Scale (VAS), which indicated the range from “no pain” to “unbearable pain” on a 100 mm horizontal line^29^. Participants were asked to determine the intensity of pain at baseline (day 0) and at 3 and 7 days after treatment started.

**(2) Ulcer size**^1^

The size of the ulcer (in mm) was recorded at baseline (day 0) and on days 3 and 7 after treatment using a calibrated dental probe with millimeter markings.

**(3) Biochemical analysis**

To reduce diurnal variation, 10 mL of unstimulated whole saliva was taken from each patient between 9:00 and 11:00 AM. For at least ninety minutes before collection, participants were told not to eat, drink, or practice oral hygiene. Patients expectorated over 10 min into a sterile, ice-chilled, 50 mL polypropylene tube using the passive drool method. To eliminate mucins and cellular debris, samples were promptly transported on ice and centrifuged at 2600 × *g* for 15 min at 4 °C. To avoid inter-assay variability, the clear supernatant was aliquoted into 1.5 mL cryovials and kept at − 80 °C until batch analysis.

**Quantification of Interleukin-2**: A commercially available solid-phase sandwich Enzyme-Linked Immunosorbent Assay (ELISA) kit (BT-LAB, Cat. No. E0094Hu) was used to measure the amount of human interleukin-2 (IL-2) in saliva. The assay was carried out precisely as directed by the manufacturer. Every standard and sample was run twice^6^.

**Assay features**: A plate coated with a human IL-2-specific capture antibody serves as the foundation for the assay’s operation. After binding to this antibody, the sample’s IL-2 is identified by a biotinylated detection antibody and Streptavidin-Horseradish Peroxidase (HRP) conjugation. Following an acid stop, the enzymatic reaction with the tetramethylbenzidine (TMB) substrate yields a color that is proportionate to the IL-2 concentration, which is measured at 450 nm.

- Range of the Standard Curve: 5–2000 ng/L (pg/mL).
- Sensitivity: 2.51 ng/L was the lowest detectable dose (assay sensitivity).
- Specificity: There has been no reported significant cross-reactivity or interference with analogous cytokines, and this kit exhibits high specificity for both natural and recombinant human IL-2.

#### Calculation

Using a 4-parameter logistic (4-PL) curve-fit regression model, the mean optical density (OD) for each of the standard concentrations (2400, 1200, 600, 300, 150, 75, and 0 ng/L) was plotted to create a standard curve. This standard curve was then used to interpolate the amount of IL-2 present in each sample. Every value is expressed in pg/mL.

**Fig. 2:**
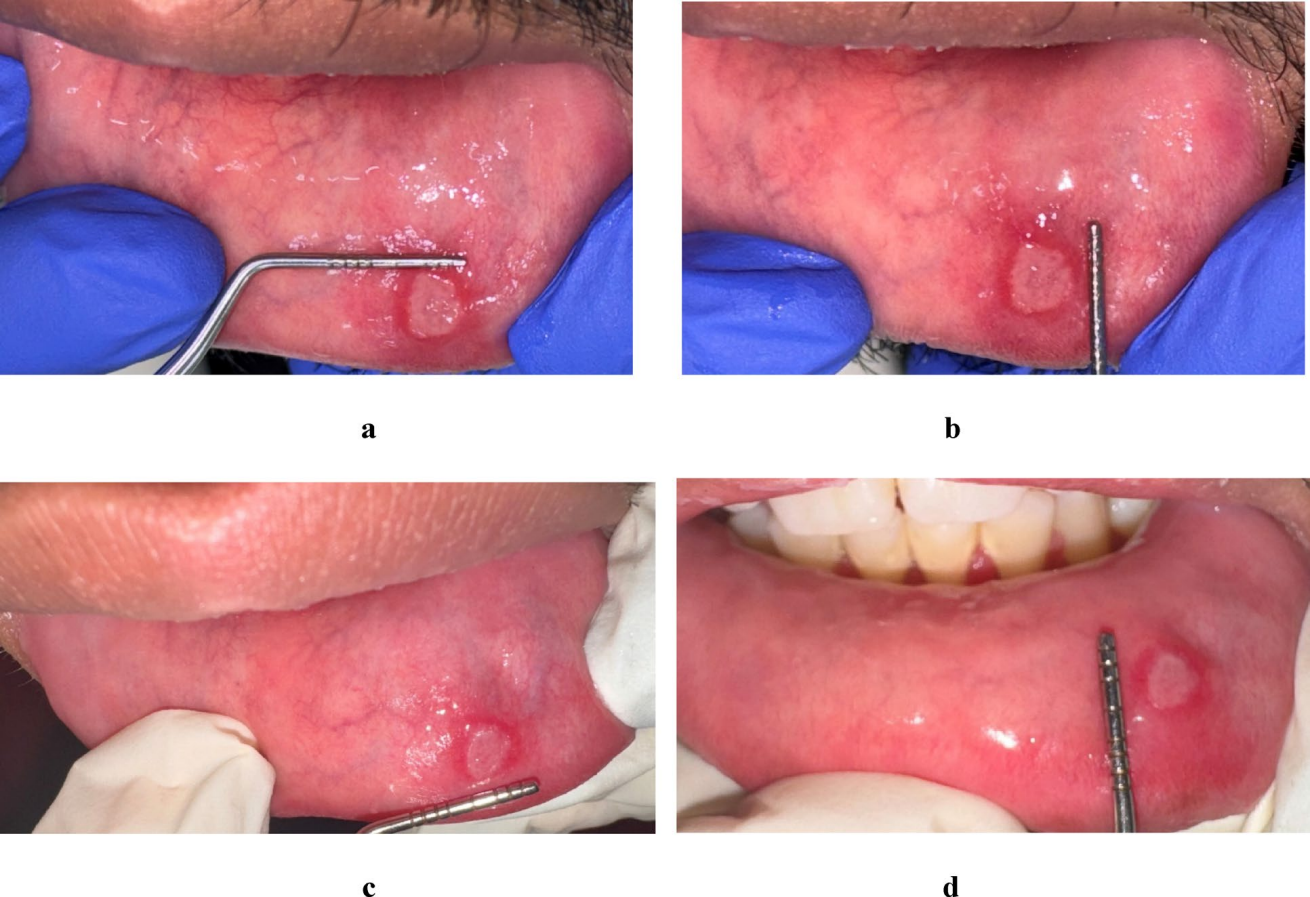

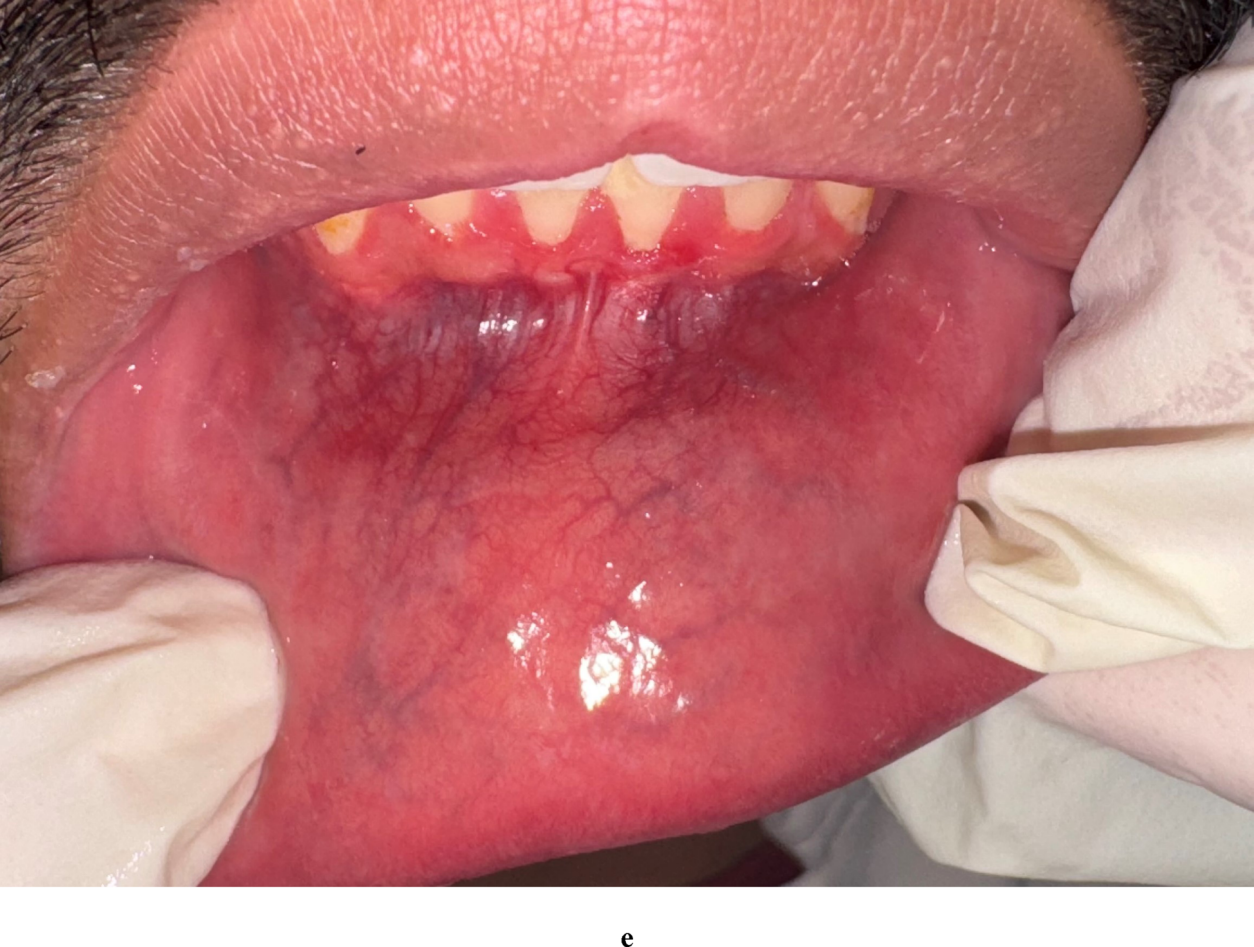
**a** and **b**: Show a round ulcer at baseline with regular edges surrounded with erythema, which is 5 × 8 in mm covered with fibrinous exudate and has not indurated base. **c** and **d**: Show the ulcer at third day 3 after treatment with EPO oral gel and it showed decrease in size and inflammation, which become 4 × 5 in mm. **e** Shows complete healing of the ulcer at day 7 after treatment by EPO oral gel.

Figure 2 shows a case of minor aphthous ulcers before and after treatment with EPO gel.

#### Intervention preparations

**Erythropoietin source and concentration**: The active medicinal ingredient was recombinant human erythropoietin (EPO) at a concentration of 4,000 IU/mL in a vial (Epogen^®^, Amgen). In both active topical formulations (mouthwash and oral gel), the final EPO concentration was 50 International Units per milliliter (IU/mL). Based on its effectiveness and safety record in earlier research on oral mucositis, this concentration was chosen^22^.

#### Preparation of study formulations

N.M., a licensed pharmacist, prepared each formulation. The same Carbopol hydrogel matrix was used to prepare the oral gel and its placebo base, and the same laboratory conditions were used to produce the erythropoietin mouthwash and placebo rinse. Before being used in clinical settings, all preparations underwent quality checks for consistency in appearance, viscosity, pH, and spreadability.

#### Preparation of EPO mouthwash

An exact amount of EPO was added to the precisely measured distilled water, which was kept under stirring at room temperature for 5 min till completely dissolved to obtain a concentration of 50 IU/ml. The pH was measured utilizing a Jenway pH meter (model 3510, UK) that was standardized before each measurement with standard pH 4, 7, and 9.2 buffer solutions, and it was adjusted to 5.5.

#### Preparation of EPO hydrogel

An accurate amount of Carbopol (3% w/w) was weighed and dissolved directly into an exactly measured amount of distilled water containing 50 IU/ml of EPO as previously mentioned and kept under stirring for 5 min till complete dissolution of the gelling agent. The pH was adjusted to 5.5 using triethanolamine under stirring till obtaining the hydrogel.

#### Preparation of placebo control

0.90% isotonic saline that had been adjusted to pH 5.5 made up the placebo mouthwash. To match their active counterparts, mint essence was used to flavor the placebo.

All final formulations- the active EPO gel, the active EPO mouthwash, and the placebo saline mouthwash went through standardized quality control testing to ensure consistency and blinding. The appearance, viscosity, pH, and taste of the two mouthwash formulations (active and placebo) were all matched. The viscosity, spreadability, pH, and appearance of the EPO gel were evaluated. To keep participants and outcome assessors blind, all formulations were administered in identical, opaque, coded containers (tubes for the gel, bottles for the mouthwashes).

#### Administration schedule

All patients across three groups were instructed to apply the interventions as per the same administration protocol (Four times a day, after the three main meals and before bedtime):

**Group I (EPO Gel)**: Patients were told to use a clean fingertip to apply a pea-sized amount (~ 0.5 g) of the gel directly onto the ulcer surface. To optimize local retention and bioavailability, they were instructed to refrain from eating or drinking for at least half an hour following application.

**Group II (EPO Mouthwash)**: Patients were told to rinse vigorously for two minutes with 10 mL of the solution before completely expectorating.

**Group III (Placebo)**: Patients used the placebo mouthwash following the same protocol as their active counterpart of the EPO mouthwash group.

#### Compliance monitoring

Throughout the study, careful attention was paid to patients’ compliance with the protocol. Structured daily diaries were given to each participant so they could note the time and frequency of each application. During the clinical evaluations on Days 3 and 7, adherence was further confirmed and strengthened by direct verbal questioning. All enrolled participants were included in the final analysis after demonstrating satisfactory compliance.

#### Adverse events monitoring

During each clinical evaluation (Day 3 and Day 7), each patient was specifically asked if they had experienced any adverse events. Both local effects (such as burning, taste changes, mucosal irritation, itching, or allergic reactions at the application site) and possible systemic effects (such as headache, lightheadedness, or nausea) were the focus of the investigations. The investigator evaluated and recorded the severity (mild, moderate, severe), duration, action taken, and relationship to the study product (not related, possibly related, probably related) for each reported adverse event.

#### Assessment of recurrence rate

For a maximum of six months, patients were monitored for recurrence by means of monthly phone calls and clinic visits. Recurrence time was defined as the clinically confirmed time between the healing of an ulcer and the start of a new ulcer episode.

#### Statistical analysis

Data were analyzed using SPSS version 23 (IBM Corp., Armonk, NY, USA) and R (lme4, survival, and emmeans packages). The primary outcome was pain reduction on the 100-mm Visual Analog Scale (VAS) at Day 7; secondary outcomes included clinical ulcer score, salivary IL-2, Effectiveness Index (EI%), and recurrence-free interval. Continuous outcomes were analyzed using linear mixed-effects models with fixed effects for *group*, *time*, and their interaction, and random intercepts for subjects. Post-hoc pairwise comparisons were Tukey-adjusted. Results are presented with effect sizes and 95% confidence intervals. Categorical EI% outcomes were compared using Chi-square tests with effect sizes (Cramér’s V, 95% CI). Recurrence data were analyzed using Kaplan–Meier survival curves with log-rank tests, and hazard ratios (HR, 95% CI) were estimated with a Cox proportional hazards model. Correlations between IL-2 and pain/clinical scores were assessed with Spearman’s rank correlation coefficients (ρ, 95% CI). A two-sided *p* ≤ 0.05 was considered statistically significant.

**Handling of missing data**: By carefully and closely monitoring patients, the study protocol was created to reduce the amount of missing data. All 60 subjects finished the study with a complete set of data points for all primary and secondary outcomes at all time intervals (baseline, Day 3, Day 7), and no participants withdrew. Consequently, there was no need for imputation techniques for missing data.

**Adjustment for multiple comparisons**: The Tukey-Kramer method was used to adjust all post-hoc analyses after significant ANOVA or mixed-model effects to control the family-wise error rate resulting from multiple pairwise comparisons between the three study groups (I vs. II, I vs. III, II vs. III) for primary and secondary outcomes (P-VAS, Ulcer Size, and IL-2). The overall significance level is kept at α = 0.05 by this cautious method. For these between-group contrasts, all p-values that are reported are Tukey-adjusted.

## Results

### Baseline characteristics

The present study was conducted on 60 subjects divided into three groups (*n* = 20). Group I comprised 14 females (70%), 6 males (30%), Group II comprised 10 females (50%), 10 males (50%), while Group III showed the same gender distribution as Group II. The mean and standard deviation (SD) values for age were 21.9 (3), 23.2 (2.8), and 24.3 (3.3) years old in the three groups, respectively.

### Clinical parameters

#### Pain visual analog scale (P-VAS)

At baseline, pain scores did not differ significantly among the three groups (*p* > 0.05). By Day 3, Group I showed significantly greater pain reduction compared with both Group II and Group III (all *p* < 0.001), and Group II also demonstrated significantly lower pain scores than Group III (*p* < 0.001). This pattern persisted at Day 5, with Group I remaining significantly superior to the other groups, followed by Group II, while Group III exhibited the least improvement (all *p* < 0.001). By Day 7, Group I achieved near-complete pain resolution and continued to demonstrate significantly lower pain scores than both Group II and Group III, with Group II also remaining significantly improved compared with Group III (all *p* < 0.001), as shown in Table 1.

**Table 1:** Comparison of mean pain-VAS scores (Mean ± SD) between the three groups at different time points.

Time	Group I (n = 20)	Group II (n = 20)	Group III (n = 20)	*P*-value	Effect size (Eta squared)
Base line	7.8 ± 0.7^a^	7.8 ± 0.6^a^	7.9 ± 0.8^a^	0.889	0.004
3 days	3.0 ± 0.6^a^	5.5 ± 0.5^b^	7.0 ± 1.0^c^	< 0.001	0.832
5 days	1.5 ± 0.8^a^	3.7 ± 1.1^b^	5.7 ± 0.8^c^	< 0.001	0.781
7 days	0.2 ± 0.4^a^	2.1 ± 0.8^b^	4.2 ± 0.7^c^	< 0.001	0.850
*P*-value	< 0.001	< 0.001	< 0.001		
Effect size (w)	0.984	0.991	0.972		

Values are expressed as Mean ± SD. Between-group comparisons were performed using one-way ANOVA with η^2^ as effect size (0.01 = small, 0.06 = medium, ≥ 0.14 = large). Within-group comparisons over time were assessed using Friedman’s test with Kendall’s W as effect size (0.1 = small, 0.3 = moderate, ≥ 0.5 = large).

**Fig. 3:**
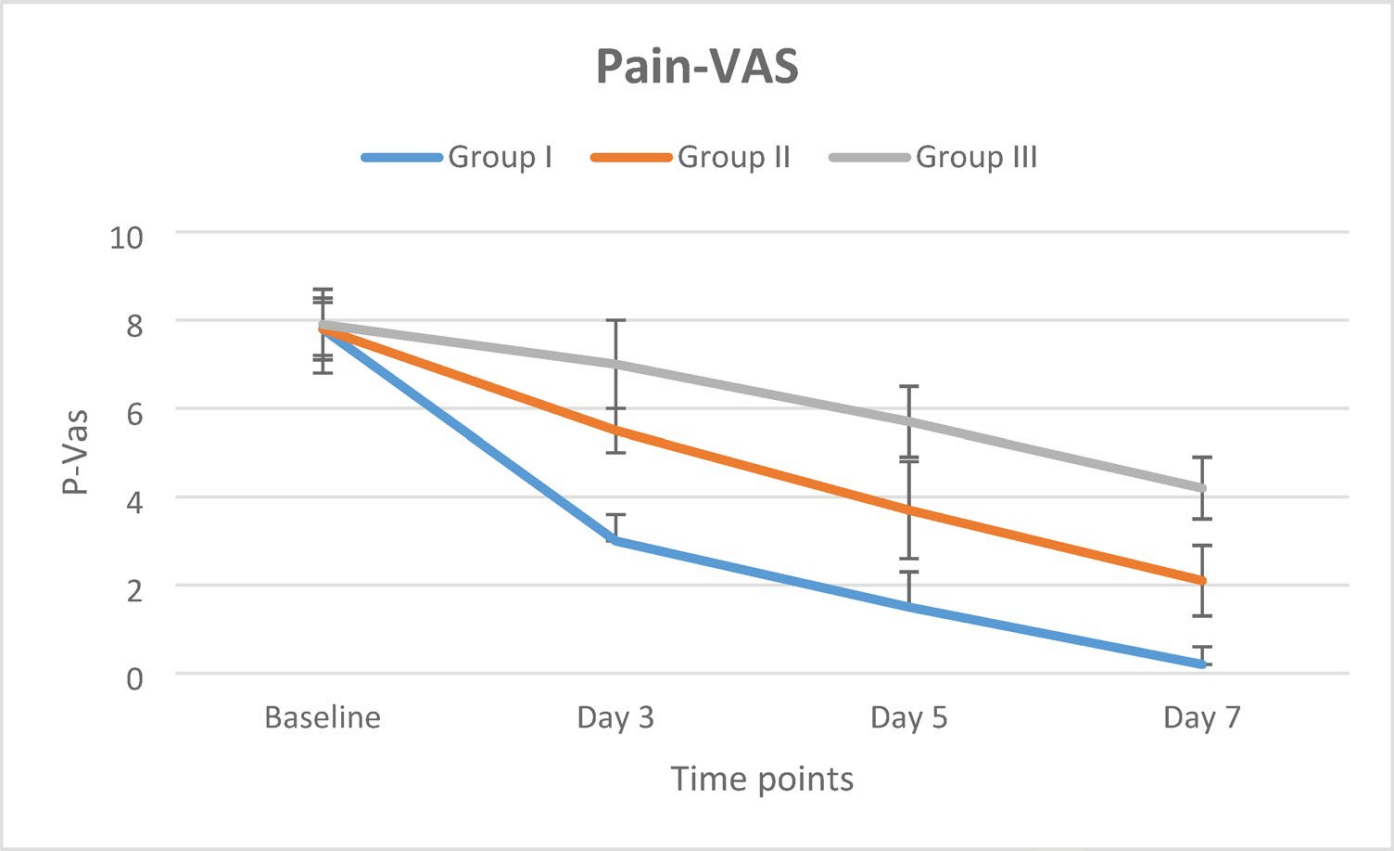
Linear chart shows change in pain intensity (VAS) scores across time in the three study groups.

Within-group analyses revealed highly significant reductions in pain from baseline to Day 7 in all groups (Friedman’s test, all *p* < 0.001), with very large effect sizes. The magnitude of pain reduction was greatest in Group I, followed by Group II, while Group III exhibited the slowest rate of improvement as in Fig. 3.

#### Clinical score (ulcer size)

At baseline, a significant difference in clinical scores was observed among the three groups (one-way ANOVA, *p* = 0.011; η^2^ = 0.146). Post-hoc analysis indicated that Group II exhibited significantly higher clinical scores than Group I, while Group III did not differ significantly from either group. By Day 7, Group I demonstrated significantly greater reduction in clinical scores compared with both Group II and Group III (both *p* < 0.001). No significant difference was detected between Groups II and III at this time point. The between-group effect size at Day 7 Within-group analyses confirmed highly significant reductions in clinical scores from baseline to Day 7 in all groups (Wilcoxon signed-rank test, all *p* < 0.001), with very large effect sizes (*r* = 0.88). The magnitude of improvement was greatest in Group I, followed by Groups II and III, as presented in Table 2.

**Table 2:** Comparison of clinical scores (Mean ± SD) between the three groups at baseline and Day 7.

Time	Group I (n = 20)	Group II (n = 20)	Group III (n = 20)	*P*-value	Effect size (Eta squared)
Before application (mm)	6.5 ± 1.0^a^	7.4 ± 0.7^b^	6.8 ± 1.0^a^,^b^	0.011	0.146
After application (mm)	0.6 ± 0.8^a^	4.1 ± 0.9^b^	4.4 ± 1.4^b^	< 0.001	0.714
*P*-value	< 0.001	< 0.001	< 0.001		
Effect size (r)	0.88	0.88	0.88		

^a^, ^b^(ANOVA/Tukey): Values with different superscripts differ significantly at *p* < 0.05 (post-hoc Tukey following one-way ANOVA). Wilcoxon signed-rank test: Used for within-group baseline vs. Day 7 comparisons. Effect size reported as r. Ulcer size was recorded as the maximum linear diameter (mm) using a calibrated periodontal probe.

#### Effectiveness index

The effectiveness index (EI) for pain reduction differed significantly among the study groups at all follow-up intervals (*p* < 0.001). At Day 3, most patients in Group I exhibited moderate or marked improvement, whereas the majority of patients in Group II and all patients in Group III showed no improvement. By Day 5, further improvement was observed in Group I, with most patients achieving marked improvement or complete healing. Group II predominantly demonstrated moderate improvement, while Group III continued to show the least favorable response. At Day 7, Group I achieved near-complete healing in the majority of cases, whereas Group II showed mainly moderate to marked improvement, and Group III remained the least improved. These findings are detailed in Table 3.

**Table 3:** Distribution of effectiveness Index (EI) categories for pain at different time points among the three groups.

Effectiveness index	Group I (n = 20)	Group II (n = 20)	Group III (n = 20)	*P*-value
Day 3				
No improvement (< 30%)	0 (0.0%)	14 (70.0%)	20 (100.0%)	< 0.001
Moderate improvement (30 to < 70%)	16 (80.0%)	6 (30.0%)	0 (0.0%)	
Marked improvement (70 to < 95%)	4 (20.0%)	0 (0.0%)	0 (0.0%)	
Healed (≥ 95%)	0 (0.0%)	0 (0.0%)	0 (0.0%)	
Mean ± SD EI (%)	**61.5 ± 7.3**	**29.4 ± 4.9**	**11.5 ± 7.8**	
Mean difference (95% CI) versus Group III	50.0 (44.2–55.8)	17.9 (12.0–23.8)	Reference	
Day 5				
No improvement (< 30%)	0 (0.0%)	2 (10.0%)	12 (60.0%)	< 0.001
Moderate improvement (30 to < 70%)	2 (10.0%)	16 (80.0%)	8 (40.0%)	
Marked improvement (70 to < 95%)	16 (80.0%)	2 (10.0%)	0 (0.0%)	
Healed (≥ 95%)	2 (10.0%)	0 (0.0%)	0 (0.0%)	
Mean ± SD EI (%)	**80.9 ± 9.9**	**52.7 ± 13.3**	**27.6 ± 9.1**	
Mean difference (95% CI) versus Group III	53.3 (46.9–59.7)	25.1 (18.9–31.3)	Reference	
Day 7				
No improvement (< 30%)	0 (0.0%)	0 (0.0%)	4 (20.0%)	< 0.001
Moderate improvement (30 to < 70%)	0 (0.0%)	8 (40.0%)	16 (80.0%)	
Marked improvement (70 to < 95%)	4 (20.0%)	12 (60.0%)	0 (0.0%)	
Healed (≥ 95%)	16 (80.0%)	0 (0.0%)	0 (0.0%)	
Mean ± SD EI (%)	**97.1 ± 5.9**	**72.9 ± 11.0**	**46.4 ± 11.0**	
Mean difference (95% CI) versus Group III	50.7 (44.9–56.5)	26.5 (20.8–32.2)	Reference	

Values are expressed as *n* (%). EI categories: No improvement (< 30%), Moderate improvement (30 to < 70%), Marked improvement (70 to < 95%), and Healed (≥ 95%). Differences among groups at each time point were assessed using Chi-square test. Significant values are in bold.

Overall, Group I consistently demonstrated the greatest pain reduction across all time points, followed by Group II, while Group III showed the lowest EI values throughout the study period.

From baseline to post-treatment, EI distributions for clinical scores (ulcer size) differed significantly among groups (*p* < 0.0001). Group I exhibited the most favorable response profile, with the highest proportion of healed and markedly improved cases, whereas Group II was characterized predominantly by moderate improvement and Group III showed a mixed pattern of moderate or no improvement. Compared with the control group, Group I demonstrated a substantially greater improvement, while Group II showed a smaller and borderline difference Table 4.

**Table 4:** Clinical scoring (ulcer size) effectiveness index (EI) from before and after treatment

Category/metric	Group I (n = 20)	Group II (n = 20)	Group III (n = 20)	*P*-value
EI categories				
No improvement (< 30%)	0 (0.0%)	2 (10.0%)	10 (50.0%)	**< 0.0001**
Moderate improvement (30–69%)	2 (10.0%)	18 (90.0%)	10 (50.0%)	
Markedly effective (70–94%)	6 (30.0%)	0 (0.0%)	0 (0.0%)	
Healed (≥ 95%)	12 (60.0%)	0 (0.0%)	0 (0.0%)	
Mean ± SD EI (%)	**91.3 ± 12.1**	44.6 ± 12.4	36.7 ± 14.5	
Mean difference versus control (95% CI) (%)	**54.6** **(46.1–63.2)**	8.0 (–0.6–16.6)	Reference	

Values represent the percentage of patients in each effectiveness index (EI%) category within each study group. EI categories were defined as: No improvement (< 30%), Moderate improvement (30–70%), Markedly effective (70–95%), and Healed (≥ 95%). Percentages are based on group sample sizes (*n* = 20 per group). Significant values are in bold.

#### Recurrence rate and recurrence-free interval

**Fig. 4:**
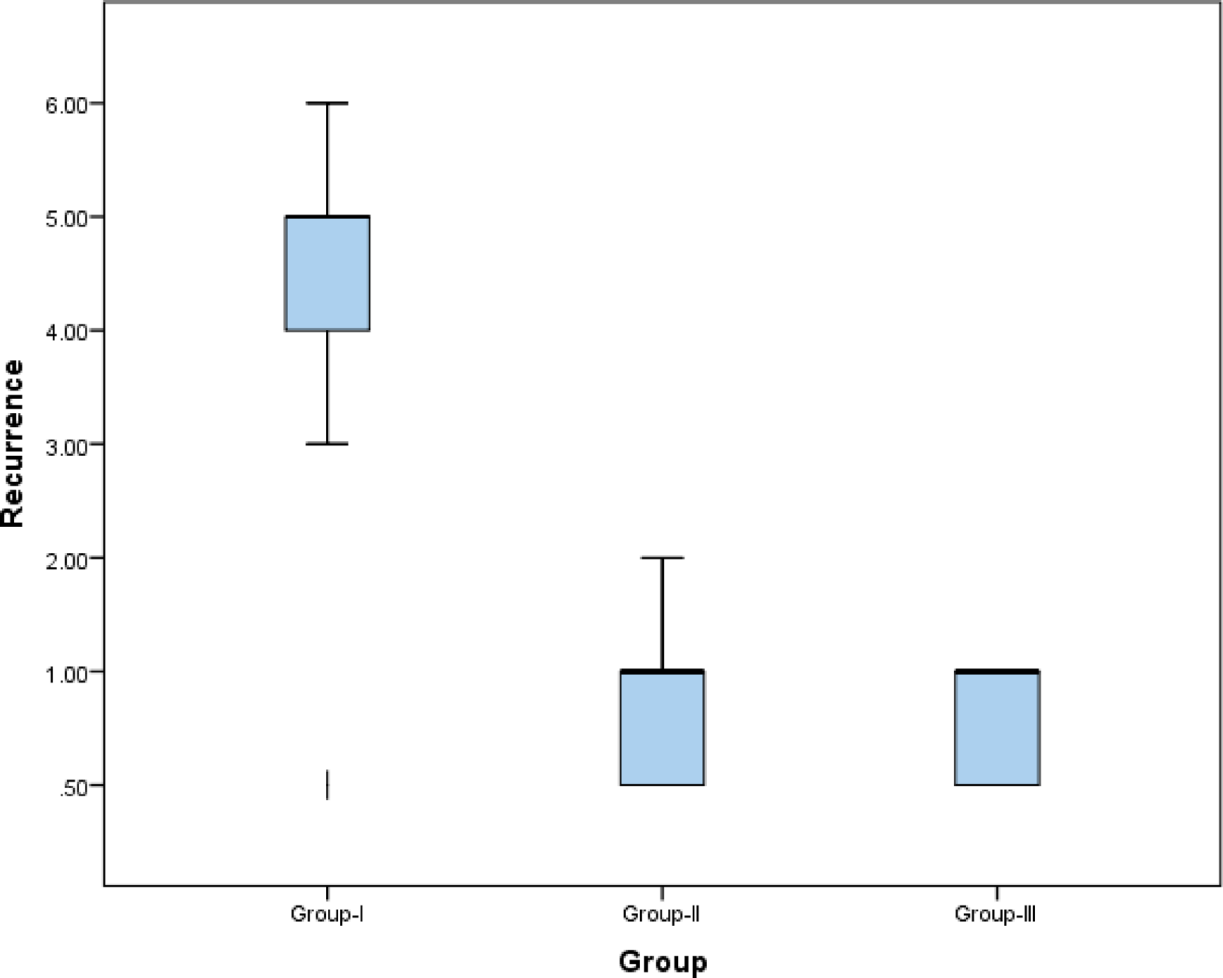
Box plot illustrating recurrence time (months) across the three study groups.

The recurrence interval differed significantly among the three groups according to the Kruskal–Wallis test (χ^2^(2) = 42.6, *p* < 0.001), with a very large effect size (ε^2^ = 0.71). Group I demonstrated a significantly longer recurrence-free interval compared with both Group II and Group III (both *p* < 0.001). No statistically significant difference was observed between Group II and Group III (*p* = 0.368). These findings are presented in Table 5; Fig. 4.

**Table 5:** Descriptive statistics and results of the Kruskal–Wallis test for comparison between recurrence rates (months) in the three groups.

Group	Median (IQR) months	Range	*P*-value^a^	Effect size (ε^2^)
Group I	5.0 (4.0–5.0)^a^	3–6	< 0.001^b^	0.72
Group II	1.0 (0.5–1.0)^b^	0.5–2	< 0.001^c^	
Group III	0.75 (0.5–1.0)^c^	0.5–1	0.041^d^	
Overall Kruskal–Wallis	χ^2^(2) = 42.6, *p* < 0.001, ε^2^ = 0.71			

^a, b, c^Indicate significant differences between medians across groups. ^b^Group I versus II: *p* < 0.001 (Holm-adjusted). ^c^ Group I versus III: *p* < 0.001 (Holm-adjusted). ^d^Group II versus III: *p* = 0.368 (n.s., Holm-adjusted).

### Biochemical parameter

#### Salivary IL-2

Salivary IL-2 levels were comparable among the three groups at baseline, with no statistically significant differences observed before application (*p* = 0.652; η^2^ = 0.015). Following application, IL-2 levels decreased significantly in all groups, with a highly significant difference detected among groups after treatment (*p* < 0.001), accompanied by a large between-group effect size (η^2^ = 0.459).

Within-group analyses demonstrated significant reductions in salivary IL-2 concentrations from baseline to post-application in all groups (all *p* < 0.001), with very large effect sizes. Although baseline levels were similar, the magnitude of IL-2 reduction differed across groups, with Group I exhibiting the most pronounced decline compared with Groups II and III. These results are presented in Table 6.

**Table 6:** Salivary IL-2 levels (pg/mL) before and after application among study groups.

Time	Group I (n = 20)	Group II (n = 20)	Group III (n = 20)	*P*-value	Effect size (η^2^)
Before application	1982.8 ± 409.6^a^	1872.3 ± 466.3^a^	1865.9 ± 465.1^a^	0.652	0.015
After application	313.7 ± 96.0^a^	596.5 ± 226.8^b^	673.2 ± 168.1^b^	< 0.001*	0.459
*P*-value	*p* < 0.001	*p* < 0.001*	*p* < 0.001*		
Effect size (Cohen’s d)	3.84	2.58	2.53		

Values are expressed as mean ± standard deviation. Different superscript letters (^a^,^b^) indicate statistically significant between-group differences at *p* < 0.05 based on post-hoc Tukey tests following one-way ANOVA. Asterisks (*) indicate statistically significant within-group differences between before and after application based on paired *t*-tests. Between-group effect sizes are reported as η^2^, and within-group effect sizes are reported as Cohen’s *d*.

### Correlations

#### IL-2 and clinical score

Spearman’s correlation analysis revealed no statistically significant associations between salivary IL-2 levels and clinical scores in any of the study groups, either before or after application. This lack of significant correlation was consistent across Groups I, II, and III. Similarly, when all participants were analyzed collectively, IL-2 levels showed weak and non-significant correlations with clinical scores at both baseline and post-application. These findings are summarized in Table 7.

**Table 7:** Descriptive statistics and results of Spearman’s correlation coefficient for correlation between IL-2 and clinical scores.

Group	Time	Correlation coefficient (ρ)	95% CI	*P*-value
Group I	Before application	− 0.297	− 0.65 to 0.17	0.204
	After application	0.000	− 0.44 to 0.44	1.000
Group II	Before application	− 0.088	− 0.51 to 0.37	0.711
	After application	0.038	− 0.41 to 0.47	0.873
Group III	Before application	0.039	− 0.41 to 0.47	0.872
	After application	− 0.242	− 0.62 to 0.23	0.296
Overall	Before application	− 0.197	− 0.46 to 0.11	0.167
	After application	− 0.144	− 0.42 to 0.14	0.314

Values are Spearman’s correlation coefficients (ρ) with 95% confidence intervals. *P*-values ≤ 0.05 were considered statistically significant.

#### Correlation between IL-2 and pain score

Spearman’s correlation analysis showed that baseline salivary IL-2 levels were generally not associated with pain scores across study groups. An exception was observed in Group I, where baseline IL-2 demonstrated a moderate, statistically significant positive correlation with pain scores on Day 5. No other significant correlations were detected at baseline, Day 3, or Day 7 in Group I. In Groups II and III, correlations between baseline IL-2 levels and pain scores were weak and non-significant at all time points. When analyzed collectively, no significant associations were observed between baseline IL-2 levels and pain scores at any assessment time as presented in Table 8.

**Table 8:** Descriptive statistics and results of Spearman’s correlation coefficient for correlation between IL-2 before application and pain scores.

Group	Time	Correlation coefficient (ρ)	95% CI	*P*-value
Group I	Baseline	– 0.181	– 0.58 to 0.28	0.446
	3 days	0.057	– 0.40 to 0.49	0.812
	5 days	0.470*	0.04 to 0.76	0.036
	7 days	0.225	– 0.24 to 0.61	0.341
Group II	Baseline	0.163	– 0.30 to 0.56	0.492
	3 days	– 0.108	– 0.53 to 0.37	0.664
	5 days	– 0.067	– 0.49 to 0.39	0.787
	7 days	– 0.237	– 0.63 to 0.24	0.312
Group III	Baseline	– 0.014	– 0.46 to 0.44	0.952
	3 days	– 0.087	– 0.51 to 0.37	0.715
	5 days	– 0.266	– 0.64 to 0.21	0.255
	7 days	– 0.272	– 0.65 to 0.21	0.246
Overall	Baseline	– 0.020	– 0.31 to 0.27	0.894
	3 days	– 0.056	– 0.34 to 0.25	0.710
	5 days	0.051	– 0.25 to 0.34	0.734
	7 days	– 0.146	– 0.42 to 0.14	0.306

Values are Spearman’s correlation coefficients (ρ) with 95% confidence intervals. *Significant at *P* ≤ 0.05.

After application, salivary IL-2 levels in Group I showed significant negative correlations with pain scores at baseline and on Day 5, indicating that greater reductions in IL-2 were associated with lower pain intensity. No significant associations were observed at Day 3 or Day 7 in this group. In contrast, Groups II and III showed no significant correlations between post-application IL-2 levels and pain scores at any time point. When all participants were analyzed together, significant negative correlations between post-application IL-2 levels and pain were observed at baseline and Day 5, while associations at Day 3 and Day 7 were not statistically significant Table 9.

**Table 9:** Descriptive statistics and results of Spearman’s correlation coefficient for correlation between IL-2 after application and pain scores.

Group	Time	Correlation coefficient (ρ)	95% CI	*P*-value
Group I	Baseline	– 0.779*	– 0.91 to − 0.51	< 0.001
	3 days	– 0.220	– 0.60 to 0.25	0.351
	5 days	– 0.543*	– 0.79 to − 0.13	0.013
	7 days	0.348	– 0.11 to 0.69	0.133
Group II	Baseline	– 0.194	– 0.59 to 0.27	0.412
	3 days	– 0.196	– 0.60 to 0.27	0.407
	5 days	– 0.057	– 0.49 to 0.41	0.814
	7 days	– 0.265	– 0.64 to 0.23	0.268
Group III	Baseline	– 0.205	– 0.60 to 0.27	0.380
	3 days	– 0.067	– 0.49 to 0.41	0.787
	5 days	– 0.330	– 0.69 to 0.15	0.152
	7 days	– 0.372	– 0.71 to 0.11	0.107
Overall	Baseline	– 0.419*	– 0.64 to − 0.16	0.002
	3 days	– 0.176	– 0.42 to 0.09	0.183
	5 days	– 0.315*	– 0.53 to − 0.07	0.013
	7 days	– 0.090	– 0.34 to 0.17	0.500

Values are Spearman’s correlation coefficients (ρ) with 95% confidence intervals. * Significant at *P* ≤ 0.05.

### Model-based outcomes and between-group differences

Pain VAS decreased significantly over time in all groups, with Group I demonstrating the greatest reduction by Day 7 compared with Groups II and III. Pairwise comparisons confirmed that Group I achieved significantly lower pain scores than both Group II and Group III, and Group II also showed significantly lower pain scores than Group III (all *p* < 0.001). Similarly, for clinical scores, Group I exhibited significantly greater improvement than both Group II and Group III. No statistically significant difference was observed between Groups II and III. The magnitude of the between-group differences was large, indicating a strong treatment effect favoring Group I. Post-treatment salivary IL-2 levels were significantly lower in Group I compared with both Group II and Group III, whereas no significant difference was detected between Groups II and III. These findings indicate a more pronounced immunomodulatory effect in Group I. Recurrence-free survival analysis demonstrated a clear advantage for Group I, which showed a significantly longer recurrence-free interval compared with both Group II and Group III. No significant difference in recurrence-free survival was observed between Groups II and III. These results are presented in Table 10.

**Table 10:** Model-based outcomes from regression analyses (LMM and Kaplan–Meier).

Outcome/timepoint	Group-I (Estimate, 95% CI)	Group-II (Estimate, 95% CI)	Group-III (Estimate, 95% CI)	Effect size
Pain VAS (Day 7)	0.20 (0.01–0.39)	2.10 (1.73–2.47)	4.20 (3.87–4.53)	η^2^ = 0.85
Clinical score (Day 7)	0.6 (0.3–0.9)	4.1 (3.7–4.5)	4.4 (4.0–4.8)	η^2^ = 0.71
Salivary IL-2 (after, pg/mL)	313.7 (250–377)	596.5 (523–670)	673.2 (599–747)	η^2^ = 0.46
Recurrence (median months)	5.0	1.0	0.75	Log-rank χ^2^=39.4–42.1

Values are model-estimated means (95% CI) from LMM; recurrence values are Kaplan–Meier medians. Effect sizes reported as η^2^ for continuous outcomes and log-rank χ^2^ for recurrence.

### Adverse occurrences results

Throughout the study period, no negative effects were reported in any of the three groups. There were no patient dropouts during the trial, and all formulations were well tolerated.

## Discussion

It is commonly known that recurrent aphthous stomatitis resolves on its own, with individual ulcers usually healing on their own in 7–14 days. The goal of treatment is to reduce the duration of painful symptoms, speed up mucosal repair, and lower recurrence rates all of which directly enhance patient quality of life rather than to change the final healing outcome. In comparison to mouthwash and a placebo, erythropoietin gel in our study significantly decreased the size of the ulcer and the intensity of pain by Day 3 and extended the time between recurrences. These results demonstrate that, despite the fact that RAS lesions ultimately go away on their own, effective treatment can alleviate suffering, lessen functional impairment (such as trouble speaking and eating), and possibly lessen the burden of recurrence.

As we know, this is the first RCT to directly compare topical EPO in mouthwash and gel formulations for RAS. The mouthwash did not differ significantly from the placebo, despite the gel formulation’s obvious superiority, highlighting the significance of the drug delivery system in mucosal healing.

The findings of this study strongly support the initial hypothesis that topically applied erythropoietin, especially in the form of gel, reveals increased effectiveness in the treatment of recurrent aphthous stomatitis rather than a mouthwash or placebo. Erythropoietin oral gel resulted in a significant decrease in the ulcer’s size, pain severity, and salivary IL-2 on day 3 of treatment. These clinical improvements are due not only to the anti-inflammatory activity of erythropoietin, as indicated by the significant down-regulation of IL-2 pro-inflammatory cytokine, but also to the exceptional formulation properties of the gel. Our system possessed increased mucoadhesion and adherence to the ulcerated surface for an extended period, which would possibly enhance local drug bioavailability and therapeutic effectiveness^30^. This highlights the relevance of drug delivery formulation for optimal clinical results for mucosal lesions like RAS and pinpoints erythropoietin gel as a prospective candidate in forthcoming therapeutic schedules.

First-line topical therapies that target pain and inflammation reduction are often the first step in the stepwise management of RAS. Topical analgesics, anti-inflammatory drugs (like amlexanox paste), and corticosteroids (like triamcinolone acetonide in orabase) are currently the standard treatments for mild-to-moderate cases. For severe, resistant disease, systemic therapies (like colchicine, pentoxifylline, or systemic corticosteroids) are used^2^. The search for safer substitutes with regenerative qualities is fueled by the well-documented local and systemic side effects of long-term corticosteroid use, including adrenal suppression, mucosal atrophy, and oral candidiasis^7^.

According to the study’s findings, topical EPO gel is a very promising therapeutic option in this field of treatment. By Day 3, its effectiveness in quickly reducing pain and ulcer size seems to be on par with or better than that of many first-line topical medications documented in the literature^8,30^. Its longest recurrence-free interval (median 5 months) may be its greatest potential benefit. This is an outcome that is rarely attained with current standard treatments, which mainly concentrate on symptomatic relief of individual episodes rather than changing the disease’s recurrence pattern. This long-lasting effect raises the possibility that EPO is influencing the local immunoinflammatory environment, which is not usually the case with traditional corticosteroids.

Therefore, EPO gel may be considered a disease-modifying agent to extend remission periods in patients who experience frequent recurrences, in addition to being a symptomatic treatment for active ulcers. It is a particularly appealing option for long-term management due to its exceptional safety profile, which eliminates the risks associated with the use of corticosteroids. To firmly establish EPO gel’s relative efficacy and place in the RAS treatment algorithm, more head-to-head trials comparing it directly with powerful topical corticosteroids are necessary.

The EPO gel formulation’s quick pain relief was the study’s main and patient-centered advantage; notable decreases in pain were noted as early as Day 3. Since the main objective of RAS management is to enhance the patient’s quality of life by minimizing the duration of severe pain and functional impairment, this prompt relief of discomfort is an essential result. We recognize that a formal patient satisfaction survey is a limitation of the current study, even though our clinical measurements accurately captured this objective improvement in pain scores. Future research would greatly benefit from including such a validated patient-reported outcome measure in order to fully capture the subjective experience and perceived benefit of treatment from the viewpoint of the patient.

A notable positive outcome of this study was the observed reduction in IL-2 levels, which is correlated with the size and pain scores following 3 and 7 days of erythropoietin treatment. Erythropoietin is recognized as a pleiotropic glycoprotein hormone that exhibits a range of anti-inflammatory, antioxidative, and wound-healing properties. These effects are facilitated through various mechanisms, including the reduction of oxygen radical concentrations, induction of lipid peroxidation, expression of intercellular adhesion molecules, leukocyte infiltration into tissues, and the inhibition of pro-inflammatory cytokine production, such as IL-2, IL-6, IL-8, IFN-γ, and TNF-α^5^. Rezazadeh et al.^31^ reported that treatment with an EPO-loaded hydrogel resulted in weight gain and a significant reduction in mucositis severity in rats with cancer treatment-induced oral mucositis, indicating the erythropoietin healing effect. More recently, Ata et al.^32^ investigated the effectiveness of EPO hydrogel in treating tongue defects in rats. After seven days of treatment, the defects were filled with newly regenerated tissue and covered by well-organized keratinized epithelium, showing multiple interdigitations with the underlying connective tissue.

 Yaghobee et al.^33^, investigated the effect of erythropoietin on wound healing by treating patients with palatal wounds using EPO gel. After 3 to 4 weeks of treatment, they reported that topical application of EPO significantly improved palatal wound healing following free gingival graft procedures. These findings prompted us to investigate the effects of EPO on recurrent aphthous stomatitis.

In the present study, a highly significant statistical difference was observed regarding the size of the ulcers among the three intervention groups following the treatment application. However, no statistically significant difference was noted between Group II (mouthwash) and the placebo group, both of which exhibited significantly higher ulcer size compared to Group I (oral gel). This finding aligns with the results reported by Hosseinjani et al.^22^, who identified a reduction and a statistically significant difference in ulcer size before and after the treatment of oral mucositis using erythropoietin mouthwash. In the present study, the size scores following treatment with erythropoietin oral gel demonstrated a greater statistically significant difference than those reported in their research.

In relation to the recurrence rate of recurrent aphthous stomatitis (RAS), the current study revealed a highly significant statistical difference among the three intervention groups. Specifically, group I (oral gel) exhibited the lowest recurrence rate, whereas groups II (mouthwash) and the control group demonstrated higher recurrence rates.

The salivary concentrations of IL-2 were evaluated, revealing an elevation in IL-2 levels at baseline across all intervention groups. These results were in agreement with Kalpana et al.^6^, in their study where there was a statistically significant increase in IL-2 in RAS patients compared to control patients.

The results of this study also align with the research conducted by Taher^5^, who reported notably higher IL-2 levels in the RAS group in comparison to the control group. In this study, a highly statistically significant difference was observed in salivary IL-2 levels among the three groups following treatment. The control group exhibited the highest levels, while group II, which received erythropoietin mouthwash, demonstrated lower values. Conversely, group I, treated with erythropoietin gel, recorded the lowest salivary IL-2 levels. This finding aligns with the study conducted by Wei et al.^34^, who reported a significant difference in salivary TNF-alpha levels, a pro-inflammatory cytokine like IL-2, in the intervention group with recurrent aphthous stomatitis (RAS) treated with prednisone, where levels decreased by day 7 compared to the control group.

Overall, there were weak and inconsistent associations between IL-2 and clinical outcomes, although there were some notable correlations, especially in the gel group. Additionally, a strong positive correlation was found between the pain visual analogue scale and the salivary IL-2 levels post-treatment, also statistically significant. Furthermore, there is a lack of existing literature providing direct comparisons to highlight any discrepancies or inconsistencies.

So, topical application of erythropoietin in the form of gel offers greater benefits and notable improvements than mouthwash application, as the gel can remain on the ulcer for a longer period, as the patient is asked to stop eating for at least 30 min after application of the gel, and also it is difficult to be washed away with salivary movements in the oral cavity. Although the mouthwash can be sprayed across a greater surface area rather than directly on the ulcer, it may reduce the drug’s effectiveness, and it cannot remain on the ulcer for an extended period because saliva can readily wash it away^8^.

The limited efficacy of the EPO mouthwash relative to the placebo can be traced back to fundamental pharmacokinetic issues of rinse-based delivery for localized mucosal lesions. First, as a solution, the mouthwash is subject to immediate and significant dilution by saliva upon introduction to the oral cavity. Second, its contact time with the ulcerated epithelium is brief, consisting only of the duration of rinsing, followed by rapid elimination through expectoration and continued salivary flow^35,36^. This transient exposure likely leads to sub-therapeutic concentrations of EPO at the target site, inadequate to achieve a sustained anti-inflammatory or regenerative effect. By contrast, the mucoadhesive gel formulation produces a protective, drug-releasing reservoir directly over the ulcer, which circumvents, in part, the effects of dilution and clearance. This critical difference in local bioavailability highlights that for a protein therapeutic like EPO intended to modulate the wound microenvironment, formulation strategy is paramount; effective delivery requires prolonged mucosal contact that a standard mouthwash cannot provide.

The EPO gel group’s median 5-month recurrence-free interval, which was less than 1 month for the placebo group and only 1 month for the mouthwash group, was a particularly noteworthy finding beyond the short-term gains. The therapy may affect the underlying immunoinflammatory dysfunction that causes RAS recurrence rather than just reducing acute ulcer symptoms, which is why this extraordinary extension of remission raises the possibility of a disease-modifying effect. This hypothesis is supported by the concurrent decrease in salivary IL-2 levels, which connects detectable immune modulation to clinical remission. This feature highlights topical EPO gel as a potentially game-changing treatment for RAS, providing both symptomatic relief and significant change in the course of the disease, provided it is confirmed in larger, longer-term studies.

As with all studies, a number of limitations exist that need to be considered in light of the encouraging results reported herein. The first is the limited number of samples (*n* = 60), which, although suitable for preliminary conclusions, does not ensure the statistical power and the incidence of the results in the general population. The homogeneity of the reference population (from a single geographical and clinical area) may also limit the generalization of findings to more heterogeneous groups. Furthermore, the very short duration of follow-up of 7 days does not allow for the long-term effects of erythropoietin on ulcer recurrence or chronic inflammation in the context of RAS to be determined. Although the level of pain and the size of the ulcer were measured using standardized instruments, these endpoints are, in part, subjective and could potentially be affected by patient perception or adherence to the treatment protocol. Additionally, the study design did not include a placebo gel control, but rather used only a saline mouthwash as a placebo. This limits the ability to differentiate whether benefits from the EPO gel were due to the active drug itself or the mucoadhesive properties of the gel base. Future studies should include a matched placebo gel to isolate the pharmacological effect of erythropoietin.

The study’s inability to accurately replicate the look and feel of every treatment form (gel, mouthwash, and saline placebo) is another drawback. Because of this, it’s possible that participants were not completely blinded, which could have led to performance bias.

Finally, even if salivary IL-2 was used as a biomarker of inflammatory activity, the study did not evaluate other cytokines, as well as other histologic markers that could contribute to an overall immunomodulatory role of EPO on oral mucosa healing.

From a digital health perspective, the combination of standardized clinical outcomes and salivary biomarker data may facilitate future integration into AI-driven models for disease monitoring, treatment response prediction, and personalized management of recurrent aphthous stomatitis^37–41^.

Future research should take several targeted approaches to address the limitations of this study. To confirm the long-term safety and efficacy of EPO gel in a wider range of populations and to better understand its influence on recurrence patterns, larger, multicenter randomized trials with longer follow-up are required. Additionally, mechanistic research utilizing a wider range of salivary cytokines and histopathological examination would aid in elucidating the immunomodulatory mechanisms by which EPO works. A matched placebo gel control should be used in subsequent trials to separate the pharmacological activity of EPO from its vehicle. To establish the relative efficacy and potential role of EPO gel in the clinical management of RAS, direct comparative studies against well-established first-line therapies, such as topical corticosteroids, are crucial.

## Conclusions

The findings of the current study indicate that the pain levels, ulcer size, and salivary levels of IL-2 for RAS are significantly reduced in the group of patients treated with erythropoietin oral gel compared to those treated with erythropoietin mouthwash. Furthermore, the recurrence rate was reduced after administration of oral erythropoietin gel. These findings indicate that erythropoietin plays a crucial role in promoting healing and alleviating pain and inflammation, while also reducing salivary IL-2 levels, which may serve as a diagnostic marker for the severity of the condition of recurrent aphthous stomatitis. Notably, the 5-month recurrence-free interval attained with EPO gel raises the possibility that this treatment may act in a way that modifies the disease, leading to a paradigm shift in the way RAS is managed.

## Data Availability

Research data supporting this publication is available from the corresponding author upon request.
